# SMAD7-Associated Glycolytic Regulation Promotes Lactate-Dependent Macrophage Phenotype Modulation in Colorectal Cancer

**DOI:** 10.3390/cancers18162719

**Published:** 2026-08-21

**Authors:** Marco Colella, Andrea Iannucci, Rachele Frascatani, Claudia Maresca, Viviana Casagrande, Vincenzo Formica, Edoardo Troncone, Andrea Divizia, Massimo Federici, Giovanni Monteleone

**Affiliations:** 1 Department of Systems Medicine, University of Rome “Tor Vergata”, 00133 Rome, Italy; marco.colella@uniroma2.it (M.C.); rachele.frascatani@gmail.com (R.F.); maresca.9595@gmail.com (C.M.); viviana.casagrande@uniroma2.it (V.C.); v.formica1@gmail.com (V.F.); troncone.edoardo@gmail.com (E.T.); federicm@uniroma2.it (M.F.); 2 Department of Biomedicine and Prevention, University of Rome “Tor Vergata”, 00133 Rome, Italy; andrea.iannucci@uniroma2.it; 3 Department of Surgery, University of Rome “Tor Vergata”, 00133 Rome, Italy; andreadivizia@live.it; 4 Gastroenterology Unit, Azienda Ospedaliera Universitaria Policlinico “Tor Vergata”, 00133 Rome, Italy

**Keywords:** TGF-β signaling, tumor microenvironment, glycolysis, hexokinase-2, lactate, tumor-associated macrophages, STAT3, immune remodeling

## Abstract

Colorectal cancer (CRC) progression is shaped by dynamic interactions between tumor-intrinsic metabolic adaptations and immune remodeling within the tumor microenvironment. In CRC, the expression of SMAD7, a classical inhibitor of TGF-β1 signaling, is increased and has been associated with tumor-associated inflammatory responses and malignant progression. In this study, we investigated the potential role of SMAD7 in regulating glycolytic metabolism and macrophage phenotype in CRC. Knockdown of SMAD7 in CRC cell lines resulted in reduced glycolytic activity, as demonstrated by decreased extracellular acidification rate, basal glycolysis, and glycolytic capacity. These metabolic changes were associated with reduced expression of the basal and IL-6- and IL-22-induced glycolytic enzyme hexokinase 2 (HK2), while glucose uptake was increased. Similar reductions in HK2 expression were observed in patient-derived CRC organoids following SMAD7 inhibition, supporting the relevance of this pathway in human tumor-derived models. Functionally, SMAD7 knockdown reduced lactate production by CRC cells and diminished the ability of tumor cell-derived conditioned medium to induce the expression of macrophage-associated immunoregulatory markers, including CD163, CD206, and ARG1. The addition of exogenous lactate restored these effects, indicating that tumor-derived lactate contributes to SMAD7-dependent control of the expression of macrophage-associated immunoregulatory markers. Analysis of human CRC transcriptomic datasets revealed positive associations between SMAD7 expression and macrophage-related signatures, including profiles associated with immunoregulatory tumor-associated macrophages. Together, these findings support a potential role for SMAD7 in controlling tumor metabolism and macrophage-associated immunoregulatory markers in CRC.

## Simple Summary

Colorectal cancer (CRC) cells are characterized by high levels of SMAD7, a protein involved in the positive control of growth and survival of cancer cells. This study aims to examine whether SMAD7 is a regulator of CRC cell metabolism and macrophage phenotype. Knockdown of SMAD7 in CRC cell lines and patient-derived organoids reduces glycolytic activity and the expression of the basal and IL-6- and IL-22-induced glycolytic enzyme hexokinase 2. Functionally, this inhibition reduces lactate production, thus diminishing the ability of CRC cells to promote the induction of immunoregulatory macrophage markers (CD163, CD206, and ARG1), an effect restored by adding exogenous lactate. Human transcriptomic data confirm a positive association between SMAD7 and tumor-associated macrophages in CRC samples. In conclusion, SMAD7 acts as a key regulator of the glycolytic pathway and metabolic–immune interface in CRC.

## 1. Introduction

Cancer progression is increasingly recognized as a consequence of complex interactions between malignant cells and the surrounding tumor microenvironment [1,2,3]. In addition to genetic alterations that drive uncontrolled proliferation, tumor cells undergo extensive metabolic adaptations that allow them to survive under conditions of nutrient limitation, hypoxia, and inflammatory stress [4,5,6,7]. These metabolic changes are not restricted to supporting tumor cell growth but can also influence communication with immune cells and contribute to the establishment of an immune-permissive microenvironment [8,9].

One of the most prominent metabolic alterations observed in cancer is increased dependence on glycolysis despite the availability of oxygen, a phenomenon commonly referred to as aerobic glycolysis or the Warburg effect [10,11]. Enhanced glycolytic activity provides tumor cells with metabolic intermediates required for biosynthesis and adaptation to environmental stress [12,13]. Importantly, glycolytic reprogramming also affects the extracellular environment through increased production of metabolites such as lactate, which can act as signaling molecules regulating immune cell function [14,15,16].

Hexokinase 2 (HK2), which catalyzes the first committed step of glycolysis, represents a critical determinant of glycolytic flux in malignant cells [17]. Increased HK2 expression has been described in several cancers, including colorectal cancer (CRC), and has been associated with tumor growth, metabolic adaptation, and resistance to cellular stress [18,19]. HK2 expression is controlled by multiple oncogenic and inflammatory pathways, including hypoxia-inducible factors, MYC, and signal transducer and activator of transcription 3 (STAT3), highlighting the close relationship between inflammatory signaling and tumor metabolism [20,21].

Beyond its metabolic functions, lactate has emerged as an important regulator of tumor immunity [22,23]. Accumulation of extracellular lactate within tumors can influence immune cell differentiation, cytokine production, and functional responses. In particular, lactate has been associated with acquisition of macrophage phenotypes characterized by tissue remodeling, immune regulation, and tumor-supportive activities [24]. Therefore, metabolic alterations in tumor cells may indirectly regulate tumor progression by shaping immune cell behavior within the tumor microenvironment.

Transforming growth factor-β1 (TGF-β1) signaling represents another major pathway involved in cancer progression and immune regulation [25,26,27,28]. While TGF-β1 may suppress early tumorigenesis, persistent pathway activation in established tumors can promote invasion, immune evasion, and therapeutic resistance [29]. SMAD7 functions as an intracellular inhibitor of canonical TGF-β signaling by limiting receptor-mediated activation of receptor-regulated Smads [30]. However, increasing evidence indicates that SMAD7 also exerts biological activities independent of canonical TGF-β regulation through interactions with multiple intracellular signaling pathways [31].

In CRC, SMAD7 expression is increased and has been associated with tumor-associated inflammatory responses and malignant progression [32,33,34,35,36]. Previous studies from our group demonstrated that SMAD7 can enhance STAT3 activation in CRC cells, suggesting that SMAD7 may participate in inflammatory signaling networks that extend beyond the regulation of TGF-β [37]. Whether SMAD7 contributes to metabolic adaptation and tumor–immune communication in CRC remains largely unexplored.

In this study, we investigated the potential role of SMAD7 in regulating glycolytic metabolism and macrophage-associated immunoregulatory markers in CRC.

## 2. Materials and Methods

### 2.1. Cell Cultures

Unless otherwise specified, all reagents were purchased from Sigma-Aldrich (Milan, Italy). The human CRC cell lines HCT116 and DLD-1 were obtained from the American Type Culture Collection (ATCC, Manassas, VA, USA) and cultured in McCoy’s 5A and RPMI-1640 media, respectively. Both media were supplemented with 10% fetal bovine serum (FBS) and 1% penicillin/streptomycin (Lonza, Verviers, Belgium). Cells were maintained at 37 °C in a humidified incubator with 5% CO_2_ and used between passages 10 and 25. For stimulation experiments, cells were left untreated or transfected with SMAD7 sense or antisense (AS) oligonucleotides for 24 h and subsequently stimulated with recombinant human IL-6 (30 ng/mL; PeproTech, London, UK) or IL-22 (15 ng/mL; R&D Systems, Minneapolis, MN, USA) for 15–30 min. Cell lines were routinely tested for Mycoplasma contamination by PCR.

### 2.2. CRC Patient-Derived Organoid Cultures

CRC patient-derived organoids were established from fresh CRC surgical samples taken from patients who underwent colon resection for sporadic CRC at the Tor Vergata University Hospital (Rome, Italy). No patients received radiotherapy or chemotherapy before undergoing surgery. The research was carried out according to the Code of Ethics of the World Medical Association (Declaration of Helsinki); informed consent was obtained, and the local ethics committee (nr. 58.23; Independent Ethics Committee of Fondazione PTV Policlinico Tor Vergata, 17/03/2023) approved the study.

All the CRC samples were used within 1–2 h following the surgical resection. Briefly, the samples were washed with ice-cold PBS, finely minced using sterile scissors, and incubated with Gentle Cell Dissociation Reagent (Cat. #100-0485, STEMCELL Technologies, Cambridge, UK). Isolated crypts were filtered through a 70 μm cell strainer and embedded in Growth Factor Reduced, Phenol Red-Free Corning Matrigel Matrix (Cat. #356231, Corning, NY, USA). Organoids were cultured in IntestiCult Organoid Growth Medium (Cat. #100-0190, STEMCELL Technologies) supplemented with penicillin/streptomycin, gentamicin (Cat. #GEN-10B, Capricorn Scientific, Ebsdorfergrund, Germany), and ROCK inhibitor Y-27632 (STEMCELL Technologies). Culture medium was replaced every 3 days [38]. For experimental analyses, established organoids were cultured for 48 h after transfection with SMAD7 sense or antisense (AS) oligonucleotide. Culture supernatants were collected for lactate quantification, whereas Matrigel domes were processed for immunohistochemistry. For this purpose, organoids were fixed for 30 min at room temperature in 2% paraformaldehyde and 0.1% glutaraldehyde in PBS, washed three times with PBS, transferred into 20% sucrose in PBS, and incubated overnight at 2–8 °C until the Matrigel domes settled [39]. The domes were then embedded in Optimal Cutting Temperature (OCT) compound and stored for cryosectioning.

### 2.3. SMAD7 Knockdown

HCT116 and DLD-1 cells were serum-starved overnight in medium containing 0.1% FBS, washed with PBS, and transfected with SMAD7 sense or AS oligonucleotides (1.5 μg/mL) for 24 h using Lipofectamine 3000 and Opti-MEM medium (Life Technologies, Milan, Italy), according to the manufacturer’s instructions [35]. The SMAD7 AS oligonucleotide used in the present study has been previously characterized and extensively validated by our group in independent studies, demonstrating efficient and reproducible inhibition of SMAD7 expression and associated phenotypic effects in relevant cellular models [34,35,36,37].

Furthermore, HCT116 and DLD-1 cells were serum-starved overnight in medium containing 0.1% FBS, washed with PBS, and transfected with CTRL siRNA or SMAD7 siRNA (Cat. #37007; Cat. #108060, Santa Cruz Biotechnology, Inc., Dallas, TX, USA) for 24 h using Lipofectamine 3000 and Opti-MEM medium (Life Technologies, Milan, Italy), according to the manufacturer’s instructions.

For organoid transfection, established CRC organoids were dissociated into single cells using TrypLE Express (Cat. #12604-013, Thermo Fisher Scientific, Waltham, MA, USA). Cells were resuspended in 450 μL of IntestiCult Organoid Growth Medium supplemented with ROCK inhibitor and mixed with 50 μL of transfection solution containing 6 μL of Lipofectamine 3000 and 6 μg/mL of SMAD7 sense or AS oligonucleotides diluted in Opti-MEM. Cells were centrifuged at 600× *g* for 1 h at 32 °C, followed by incubation at 37 °C for 3 h [38]. Cells were then resuspended in Matrigel and cultured until use.

### 2.4. Seahorse Metabolic Analysis

Extracellular acidification rate (ECAR) was measured using a Seahorse XFp Analyzer (Agilent Technologies, Santa Clara, CA, USA). HCT116 (8 × 10^4^ cells/well) and DLD-1 (7 × 10^4^ cells/well) cells were seeded into Seahorse XFp 8-well plates and treated as indicated. Twenty-four hours later, cells were washed twice and incubated for 45 min at 37 °C in Seahorse Assay Medium supplemented with 25 mM glucose and 2 mM glutamine. ECAR was measured under basal conditions and following sequential injections of oligomycin (1.5 μM), 2,4-dinitrophenol (0.75 mM), rotenone (0.5 μM) plus antimycin A (0.5 μM), and 2-deoxy-D-glucose (50 mM) [40]. Basal glycolysis was calculated as basal ECAR minus ECAR after 2-deoxy-D-glucose treatment, whereas glycolytic capacity was calculated as ECAR after oligomycin minus ECAR after 2-deoxy-D-glucose. At the end of each assay, DNA content was quantified by propidium iodide fluorescence after Triton X-100 permeabilization to normalize ECAR values to cell number.

### 2.5. Glucose Uptake and Lactate Production

Glucose uptake was assessed using a fluorescent glucose uptake assay kit (ab235976, Abcam, Cambridge, UK). Cells (2 × 10^4^ cells/well) were seeded into 96-well plates and treated as indicated. Following treatment, 2-NBDG was diluted 1:100 in glucose-free culture medium and incubated with cells for 3 h. Fluorescence was measured according to the manufacturer’s instructions. Lactate concentrations in culture supernatants were determined using a Lactate Detection Assay Kit (Cat. MAK329) following the manufacturer’s protocol.

### 2.6. THP-1 Cell Cultures

Human THP-1 monocytic cells (ATCC) were cultured in RPMI-1640 medium supplemented with 10% FBS, 1% penicillin/streptomycin/L-glutamine (Lonza), and 0.05 mM 2-mercaptoethanol (Gibco, Carlsbad, CA, USA). Cells were maintained at 37 °C in a humidified atmosphere containing 5% CO_2_ at a density between 2 × 10^5^ and 8 × 10^5^ cells/mL. To generate resting macrophages (M0), THP-1 cells were treated with 100 ng/mL phorbol 12-myristate 13-acetate (PMA) for 30 h, followed by a 48 h resting period before subsequent treatments [41,42].

To establish the co-culture, transfected CRC organoids, prepared as described above, were embedded in Matrigel domes and seeded into Transwell permeable inserts (0.4 μm pore size) containing complete IntestiCult medium. Differentiated THP-1 cells (5 × 10^2^ cells) were seeded in the bottom of a 24-well plate. The Transwell inserts were then placed into the wells containing THP-1 cells, allowing paracrine cross-talk through the shared culture medium without direct cell-cell contact [43,44].

### 2.7. Immunohistochemistry

Cryosections of CRC patient-derived organoids were fixed in 4% paraformaldehyde for 5 min at RT, washed three times with PBS, and incubated with 3% hydrogen peroxide in methanol for 5 min to quench endogenous peroxidase activity. Sections were incubated overnight at 4 °C with antibodies against SMAD7 (1:100; SAB Biotherapeutics, Cat. #37036) or HK2 (1:100; Abcam, Cat. #ab209847). Immunostaining was visualized using the MACH4 Universal HRP-Polymer Detection Kit with DAB chromogen (Biocare Medical, Pacheco, CA, USA), according to the manufacturer’s instructions [45]. Images were acquired using a Leica DMI4000 B microscope equipped with Leica Application Suite software (version 4.6.2) using 5× and 20× objectives.

### 2.8. Western Blotting

Cells were lysed on ice in RIPA buffer containing 10 mM Tris-HCl (pH 8.0), 140 mM NaCl, 0.1% SDS, 0.1% sodium deoxycholate, 1% Triton X-100, 1 mM EDTA, 0.5 mM EGTA, protease inhibitor cocktail (Roche), and phosphatase inhibitors [35,46]. Lysates were clarified by centrifugation at 15,000 rpm for 30 min. Equal amounts of protein were separated by SDS-PAGE, transferred to membranes, and incubated with antibodies against SMAD7 (1:1000, Cat. #MAB2029, R&D Systems), HK2 (1:1000, Cat. #2867, Cell Signaling Technology), p-STAT3^(Y705)^ (1:1000, Cat. #9145, Cell Signaling Technology), STAT3 (1:1000, Cat. #12640, Cell Signaling Technology), or β-actin (1:5000, Cat. #A544, Sigma-Aldrich). Membranes were subsequently incubated with HRP-conjugated anti-rabbit or anti-mouse secondary antibodies (1:20.000; Cat. # P0448; Cat. # P0161, Life Technologies), and immunoreactive bands were detected by enhanced chemiluminescence (All uncropped images of Western blotting are available in the Supplementary Materials).

### 2.9. Real-Time PCR

Total RNA was extracted using the PureLink RNA Mini Kit (Thermo Fisher Scientific). Reverse transcription and real-time PCR (RT-PCR) were performed as previously described [47,48]. PCR cycling conditions consisted of an initial denaturation at 95 °C for 1 min, followed by annealing at 59 °C for 30 s and extension at 72 °C for 30 s. Primers were designed to span exon-exon junctions to avoid amplification of genomic DNA. Gene expression was normalized to *B2M* for CRC cells and organoids and *GAPDH* for THP-1 cells using the 2^−ΔΔCt^ method [49]. All reactions were performed in triplicate.

Primer sequences were as follows:

*SMAD7* Fw 5′-GCCCGACTTCTTCATGGTGT-3′, Rev 5′-TGCCGCTCCTTCAGTTTCTT-3′;
*HK2* Fw 5′-ATTGTCCAGTGCATCGCGGA -3′, Rev 5′-AGGTCAAACTCCTCTCGCCG-3′;
*B2M* Fw 5′-GTGGCCTTAGCTGTGCTC-3′, Rev 5′-AGAAAGACCAGTCCTTGCTG-3′;
*GAPDH* Fw 5′-GCTCTCTGCTCCTCCTGTTC-3′, Rev 5′-ACGACCAAATCCGTTGACTC-3′;
*CD163* Fw 5′-CCAGAAGGAACTTGTAGCCAC-3′, Rev 5′-CAGGCACCAAGCGTTTTGAG-3′;
*CD206* Fw 5′-AGCCAACACCAGCTCCTCAA-3′, Rev 5′-CAAAACGCTCGCGCATTGTC -3′;
*ARG1* Fw 5′-TCATCTGGGTGGATGCTCAC-3′, Rev 5′-GAGAATCCTGGCACATCGG-3′;
*IL-10* Fw 5′-AGCCTTGTCTGAGATGTCC -3′, Rev 5′-CCTTGCTCTTGTTTTCACAG-3′;
*IL-1*β Fw 5′-AGAATGACCTGAGCACCTTC-3′, Rev 5′-GCACATAAGCCTCGTTATCC-3′;

### 2.10. Analysis of Macrophage-Associated Signatures in Human CRC Transcriptomic Datasets

The TIMER3 web platform was used to investigate the association between *SMAD7* gene expression and estimated infiltration levels of total macrophages and M2 macrophages in CRC samples [50]. The specific cancer cohort analyzed was obtained from the Cancer Genome Atlas Colon Adenocarcinoma (TGCA-COAD), comprising 456 tumor samples profiled using bulk RNA sequencing. The correlation coefficient (ρ) and corresponding p-value were reported for each analysis. The TIMER3-derived immune infiltration estimates represent computational predictions from bulk tumor transcriptomic data and were therefore interpreted as correlative measures rather than direct measurements of macrophage abundance or phenotype.

### 2.11. Statistical Analysis

Statistical analyses were performed using GraphPad Prism 6 (GraphPad Software, version 6.07 for Windows, San Diego, CA, USA). Comparisons between two groups were conducted using the unpaired two-tailed Student’s *t*-test. RT-PCR data are presented as mean ± standard deviation. For the TIMER3 analysis, the association between SMAD7 expression and macrophage-related signatures was assessed using Spearman’s rank correlation analysis, with correlation coefficients (ρ) and corresponding *p*-values reported. A *p*-value < 0.05 was considered statistically significant. The survival curves were generated using the Kaplan–Meier method to evaluate the prognostic value of *SMAD7* expression. Patients were stratified into high and low *SMAD7* expression groups based on the median expression level (50th percentile cutoff).

The log-rank test was used to compare cumulative survival between the two expression groups. Hazard ratios (HRs) and corresponding 95% confidence intervals (CIs) were calculated using a univariate Cox proportional hazards regression model. All statistical tests were two-tailed, and a *p* < 0.05 was considered statistically significant. Follow-up time was evaluated up to 200 months.

## 3. Results

### 3.1. SMAD7 Regulates Glycolytic Activity and HK2 Expression in Colorectal Cancer Cells

**Figure 1:**
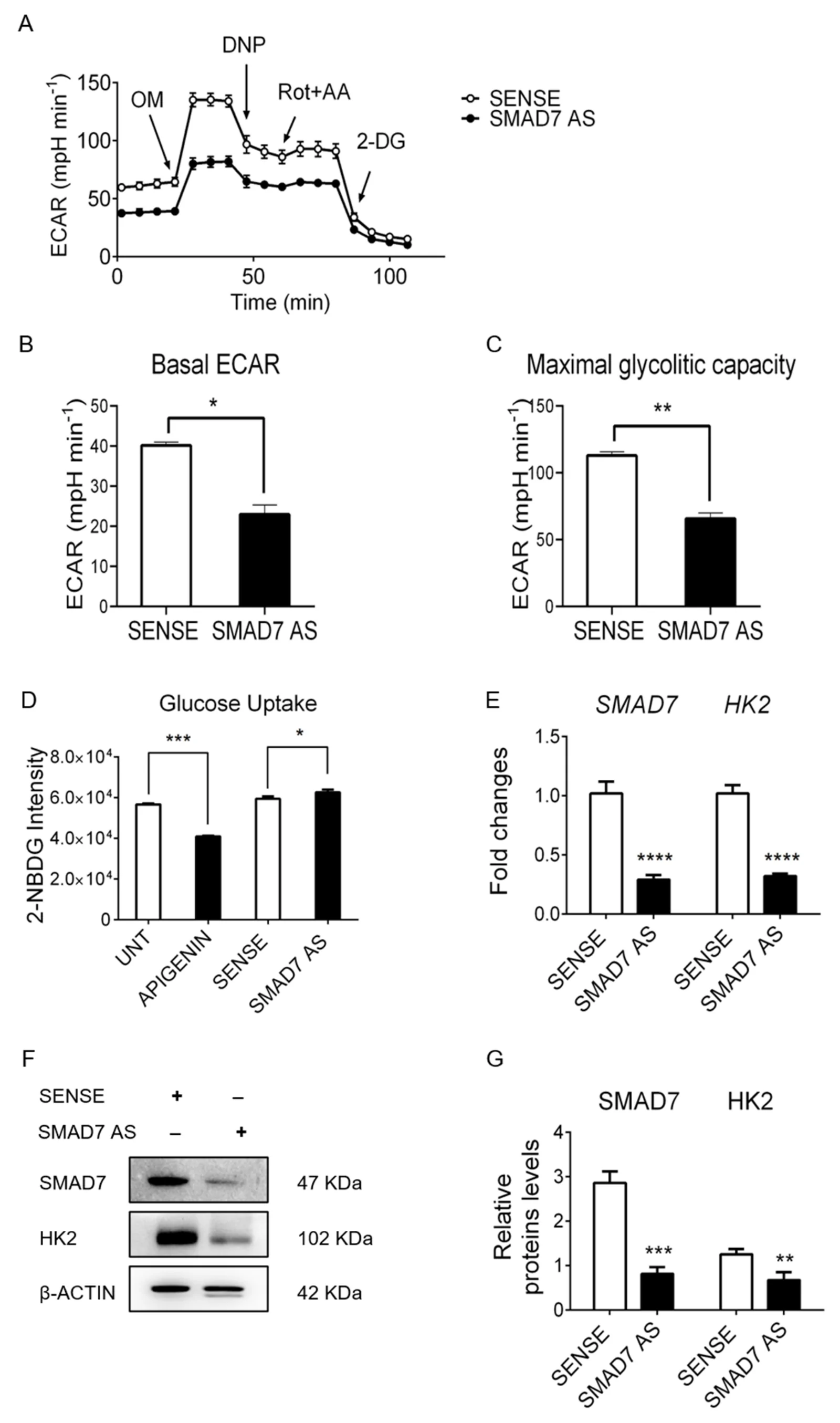
SMAD7 knockdown in HCT116 cells regulates glycolytic activity and the expression of HK2: (**A**) Extracellular acidification rates (ECARs) were measured using a Seahorse assay. (**B**,**C**) Values quantifying metabolic parameters, basal glycolysis, and maximal glycolytic capacity are reported. OM: oligomycin; DNP: 2,4-dinitrophenol; Rot: rotenone; AA: antimycin A; 2-DG: 2-deoxyglucose. Values show the mean ± SD of three independent experiments. Differences were analyzed using a two-tailed Student’s *t*-test (* *p* < 0.05, ** *p* < 0.01). (**D**) Glucose uptake evaluated in SMAD7-deficient cells. Values show the mean ± SD of three independent experiments. Differences were analyzed using a two-tailed Student’s *t*-test (* *p* < 0.05, *** *p* < 0.001). (**E**) HCT116 cells were transfected with either SMAD7 sense or AS for 18 h, and *SMAD7* and *HK2* mRNA transcripts were evaluated by real-time polymerase chain reaction. Levels were normalized to *B2M*. Values show the mean ± SD of three independent experiments. Differences were analyzed using a two-tailed Student’s *t*-test (**** *p* < 0.0001). (**F**) Cells were transfected with either SMAD7 sense or AS for 24 h, and SMAD7, HK2, and β-Actin proteins were analyzed by Western blotting. Panel (**G**) shows the quantitative analysis of SMAD7 and HK2 as evaluated by densitometric scanning of Western blots. Levels were normalized to β-actin. Values indicate the mean ± SD of three independent experiments; differences were analyzed using a two-tailed Student’s *t*-test (** *p* < 0.01, *** *p* < 0.001).

To investigate whether SMAD7 contributes to metabolic regulation in CRC, HCT116 and DLD-1 cells were transfected with SMAD7 AS or sense oligonucleotides and analyzed using real-time extracellular flux measurements. SMAD7 knockdown resulted in reduced ECAR compared with control cells, indicating decreased glycolytic activity following SMAD7 inhibition (Figure 1A). Quantification of metabolic parameters demonstrated reduced basal ECAR (Figure 1B) and maximal glycolytic capacity (Figure 1C) in SMAD7-deficient cells. Similar metabolic alterations were observed in DLD-1 cells (Figure S1A–C).

To explore the mechanism underlying the reduced glycolytic activity, glucose uptake was assessed following SMAD7 inhibition. SMAD7-deficient cells displayed increased glucose uptake compared with control cells, whereas treatment with the glucose transport inhibitor apigenin markedly reduced glucose uptake (Figure 1D). These findings suggest that reduced glycolytic flux following SMAD7 depletion is unlikely to be caused by impaired glucose availability and may instead involve regulation of downstream glycolytic processes.

Because HK2 represents a critical regulatory enzyme controlling glucose utilization and glycolytic flux, HK2 expression was subsequently examined. Real-time PCR and Western blotting analyses demonstrated that SMAD7 inhibition reduced *HK2* mRNA and protein expression in HCT116 cells (Figure 1E–G). Efficient SMAD7 knockdown was confirmed at both transcript and protein levels. Comparable reductions in HK2 expression were observed in DLD-1 cells following SMAD7 knockdown (Figure S1D–G). To determine whether the reduction in HK2 was specific to the antisense approach, we independently silenced SMAD7 using a commercially available siRNA. SMAD7 siRNA reduced SMAD7 expression and was accompanied by a significant reduction in HK2 expression, reproducing the phenotype observed with SMAD7 antisense oligonucleotide treatment in HCT116 and DLD-1 cells (Figure S2). Together, these findings indicate that SMAD7 contributes to the maintenance of glycolytic activity in CRC cells and identify HK2 as a potential metabolic effector associated with SMAD7 expression.

### 3.2. SMAD7 Inhibition Reduces HK2 Expression in Patient-Derived Colorectal Cancer Organoids

**Figure 2:**
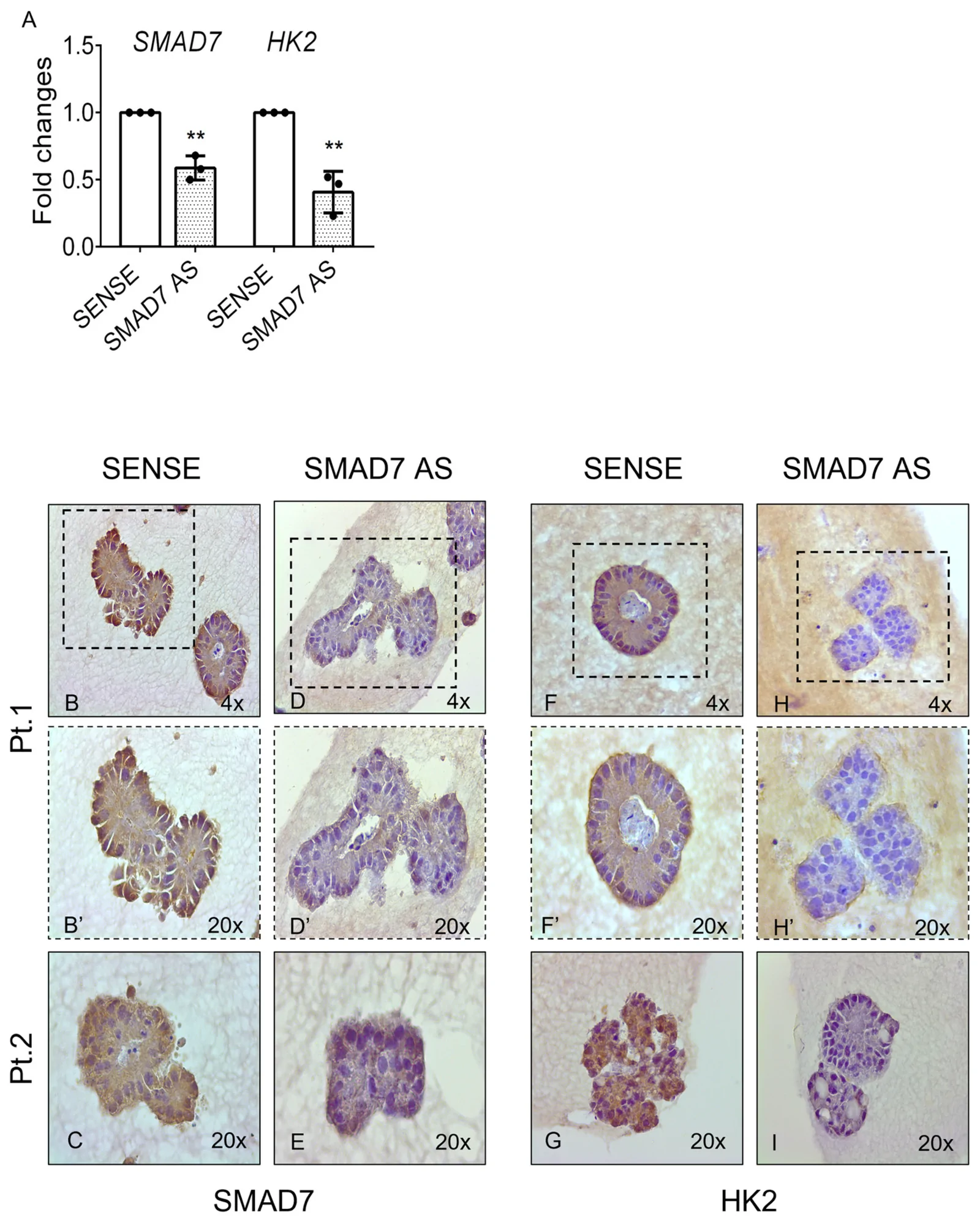
SMAD7 knockdown reduces HK2 expression in patient-derived colorectal cancer organoids: (**A**) Colorectal cancer organoids were transfected with either SMAD7 sense or AS for 24 h, and *SMAD7* and *HK2* mRNA transcripts were evaluated by real-time polymerase chain reaction. Levels were normalized to *B2M*. Values show the mean ± SD of three independent experiments. Differences were analyzed using a two-tailed Student’s *t*-test (** *p* < 0.01). (**B**) Colorectal cancer organoids were transfected with either SMAD7 sense or AS for 48 h. Representative IHC images showing SMAD7 levels (**B**–**E**) and HK2 (**F**–**I**) staining (5×; 20×). The figure is representative of three separate experiments in which similar results were obtained.

To evaluate whether SMAD7-dependent regulation of HK2 expression could be observed in a more physiologically relevant tumor model, experiments were performed using patient-derived CRC organoids. CRC organoids were treated with SMAD7 AS or control oligonucleotides, and SMAD7 expression was assessed by real-time PCR. SMAD7 inhibition reduced *SMAD7* and *HK2* RNA expression (Figure 2A). Immunohistochemical analysis of organoid sections confirmed reduced SMAD7 protein expression following SMAD7 AS treatment, which was accompanied by decreased HK2 protein expression compared with control organoids (Figure 2B–I).

### 3.3. SMAD7 Regulates STAT3-Inducing Cytokine-Mediated HK2 Expression

**Figure 3:**
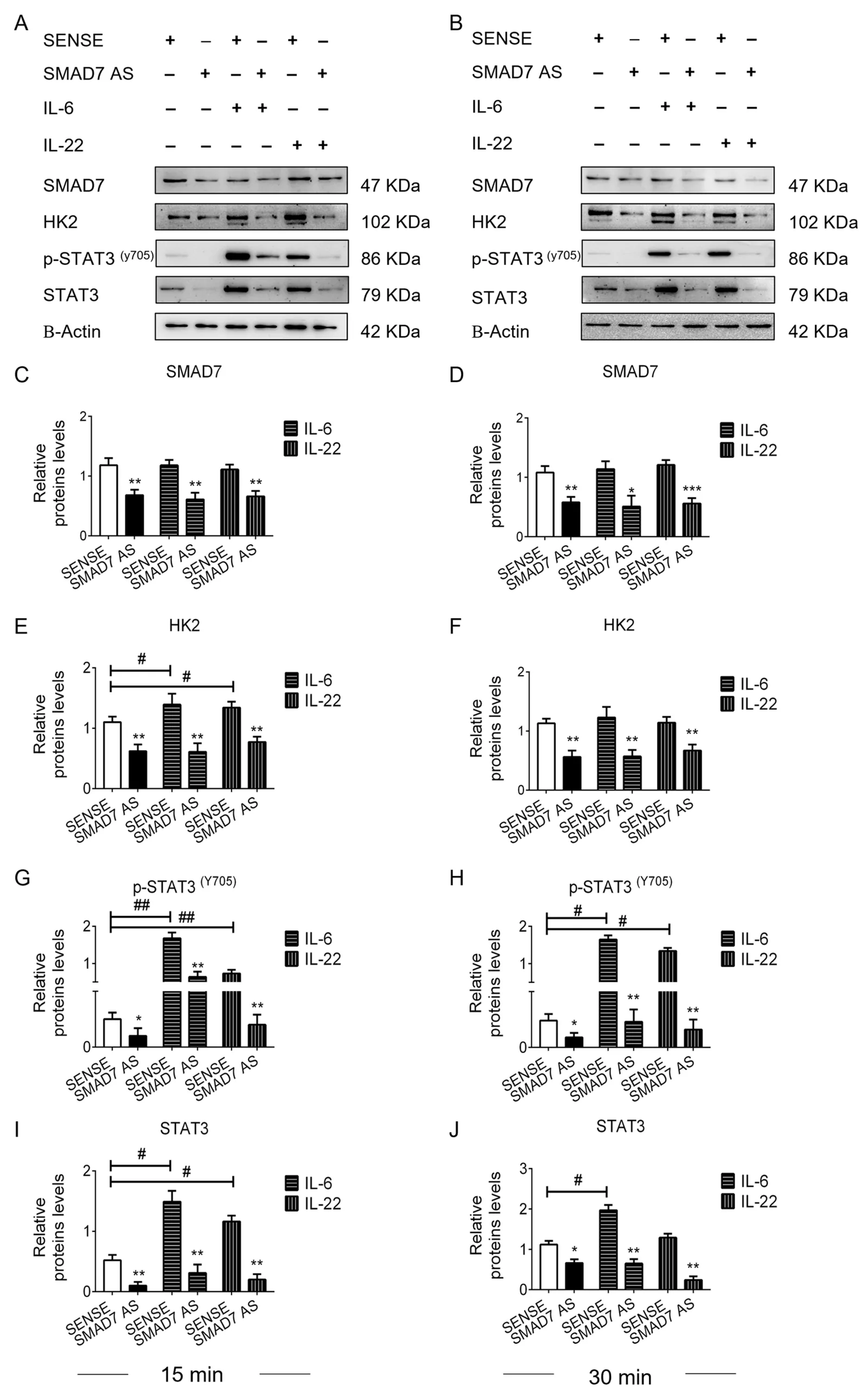
SMAD7 controls HK2 expression and STAT3 activation: (**A,B**) Cells were transfected with either SMAD7 sense or AS for 24 h and then either left untreated or stimulated with IL-6 or IL-22 for 15 or 30 min. SMAD7, HK2, p-STAT3^(y705)^, STAT3, and β-Actin proteins were analyzed by Western blotting. Panel (**C**–**J**) shows the quantitative analysis of SMAD7, HK2, p-STAT3^(Y705),^ and STAT3 as evaluated by densitometric scanning of Western blots. Levels were normalized to β-actin. Values indicate the mean ± SD of three independent experiments. Differences were analyzed using a two-tailed Student’s *t*-test (* *p* < 0.05, ** *p* < 0.01, *** *p* < 0.001; ^#^ *p* < 0.05, ^##^ *p* < 0.01).

In CRC cells, SMAD7 modulates STAT3 signaling activity [35,37]. Because STAT3 represents an important regulator of inflammatory responses and tumor-associated metabolic pathways [51,52], we investigated whether SMAD7 influences cytokine-induced HK2 expression. HCT116 cells were transfected with SMAD7 AS or control oligonucleotides and stimulated with the STAT3-activating cytokines IL-6 or IL-22 for 15 or 30 min. In control cells, stimulation with either cytokine increased p-STAT3^(Y705)^, STAT3 (Figure 3), and *HK2* transcripts (Figure S3A,B). SMAD7 inhibition reduced basal p-STAT3^(Y705)^ and HK2 expression and significantly attenuated cytokine-driven p-STAT3^(Y705)^ and HK2 induction (Figure 3A,B). Western blot analysis confirmed reduced SMAD7 protein expression following SMAD7 AS treatment and demonstrated decreased HK2 protein expression in SMAD7-deficient cells under both unstimulated and cytokine-stimulated conditions (Figure 3C–J). Comparable effects were also seen in DLD-1 cells (Figures S3C,D and S4).

### 3.4. SMAD7 Regulation Influences Lactate Production and Macrophage-Associated Inflammatory and Immunoregulatory Gene Expression

**Figure 4:**
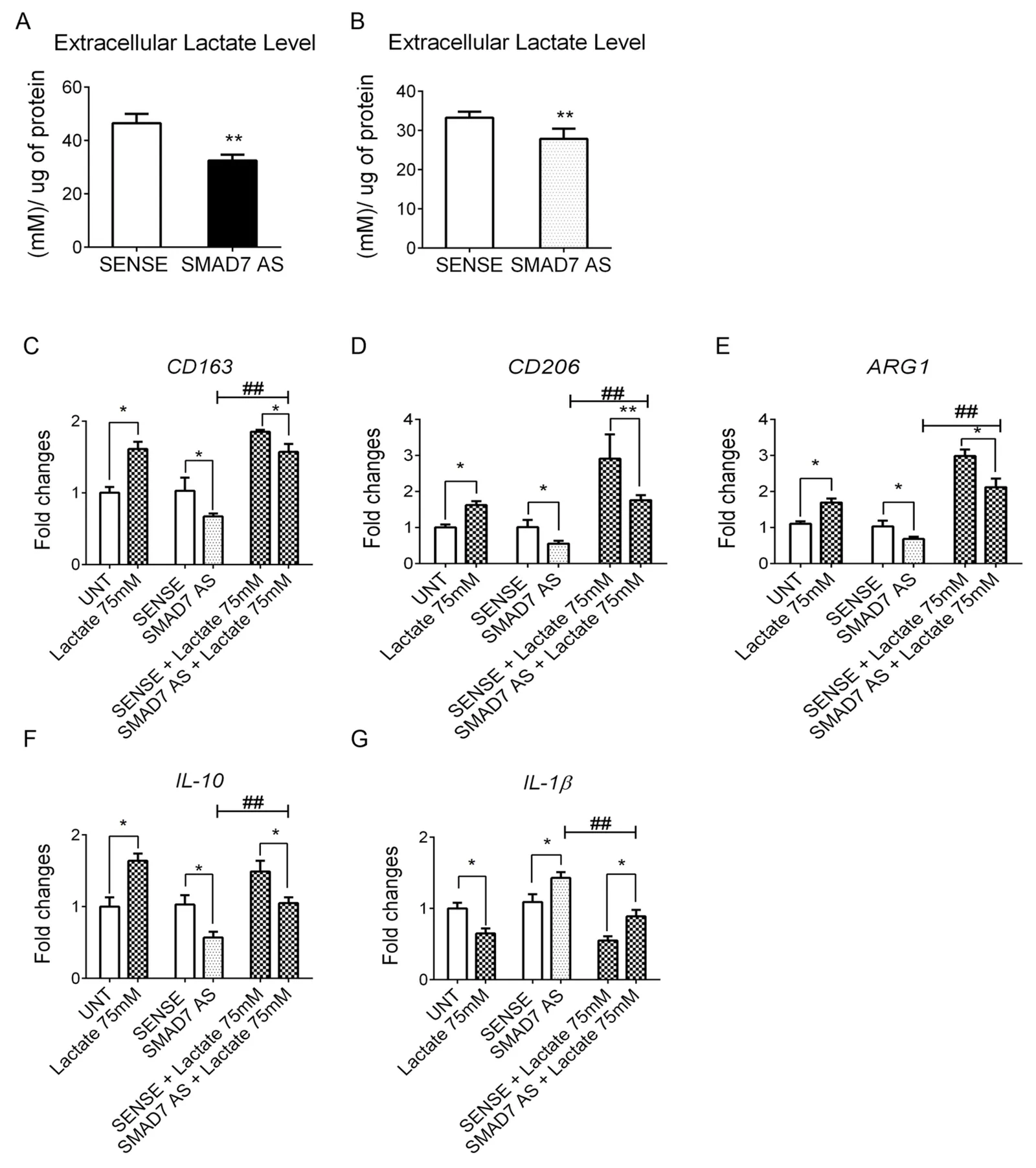
SMAD7 regulation influences lactate production and macrophage phenotype: (**A,B**) Quantification of extracellular lactate levels in the HCT 116 cell line and in colorectal cancer organoids, respectively. (**C**–**G**) THP-1 cells were cultured with medium from colorectal cancer organoids transfected with either SMAD7 sense or AS for 24 h and treated or not with exogenous lactate. *CD163*, *CD206, ARG1, IL-10*, and *IL-1β* RNA transcripts were evaluated by real-time polymerase chain reaction. Levels were normalized to *GAPDH*. Differences were analyzed using a two-tailed Student’s *t*-test (* *p* < 0.001, ** *p* < 0.0001; ^##^ *p* < 0.01).

Because glycolytic metabolism affects the production of extracellular metabolites capable of influencing immune cells [15], we investigated whether SMAD7-dependent metabolic changes alter lactate production and tumor–macrophage communication. SMAD7 inhibition resulted in reduced lactate production by HCT116 cells compared with control cells (Figure 4A) and in DLD-1 cells (Figure S1H). Similar reductions in lactate production were observed in patient-derived CRC organoids following SMAD7 inhibition (Figure 4B), consistent with reduced glycolytic activity.

To determine whether lactate could influence macrophage-associated regulatory molecules, differentiated THP-1 cells were cultured with conditioned medium from patient-derived CRC organoids following SMAD7 inhibition and treated with or without exogenous lactate. Lactate exposure increased the expression of macrophage-associated immunoregulatory markers, including *CD163*, *CD206*, and *ARG1*, as well as *IL-10*, while reducing *IL-1β* expression (Figure 4C–G). THP-1 cells cultured with conditioned medium from SMAD7-deficient organoids exhibited reduced expression of *CD163*, *CD206*, *ARG1*, and *IL-10*, together with increased *IL-1β* expression, compared with cells cultured with conditioned medium from control organoids (Figure 4C–G). The addition of exogenous lactate restored the expression of these markers in THP-1 cells cultured with conditioned medium from SMAD7-deficient organoids (Figure 4C–G).

### 3.5. SMAD7 Expression Correlates with Macrophage-Associated Signatures in Colorectal Cancer Datasets

**Figure 5:**
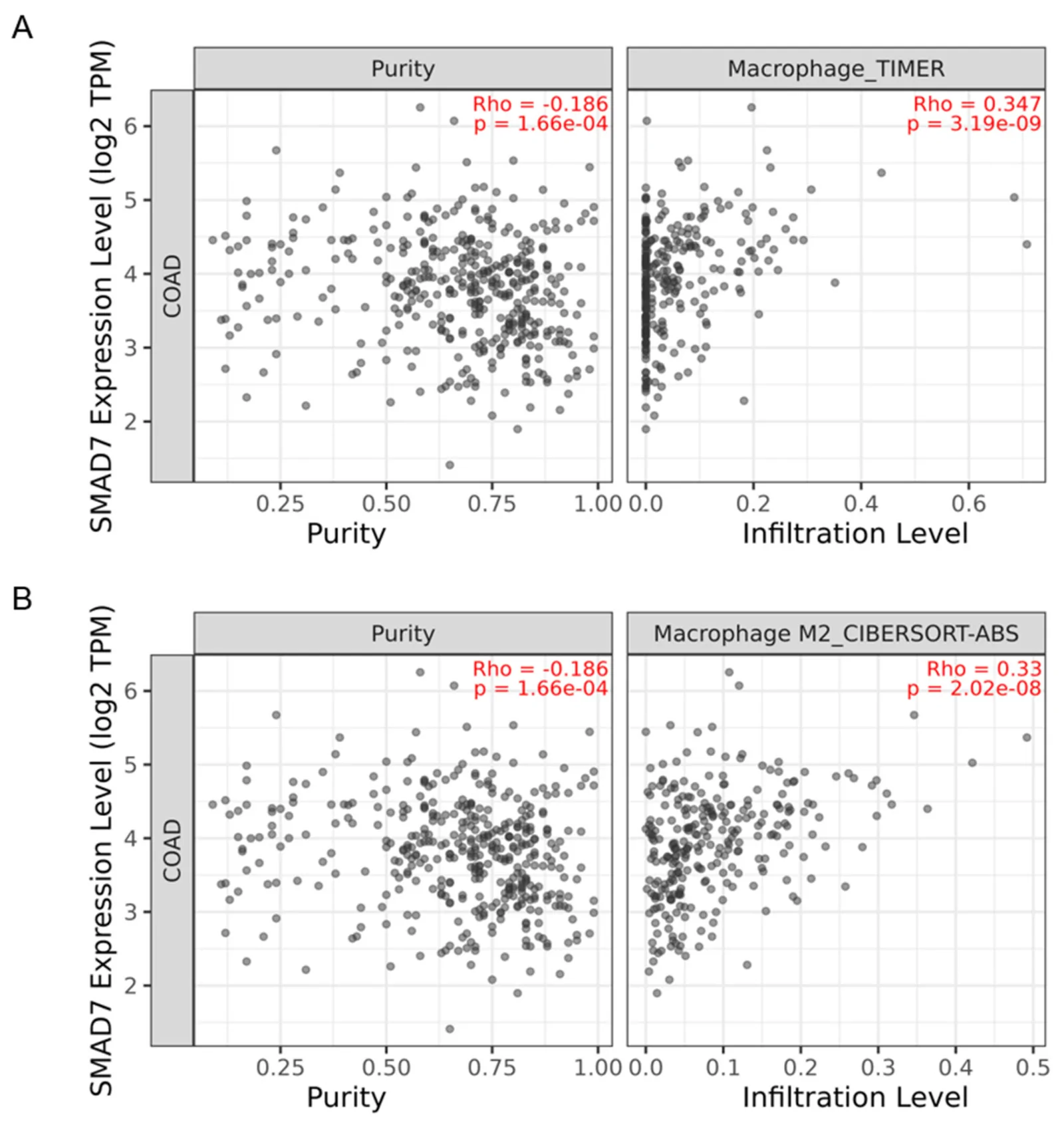
Correlation between the levels of SMAD7 and tumor-associated macrophages. Scatterplots of the correlations between SMAD7 expression and total macrophages or type 2 macrophages in colorectal cancer samples using the TIMER3 database. (**A**) Correlation between SMAD7 expression and tumor purity (**left**), and overall macrophage infiltration level calculated via the TIMER algorithm (**right**); (**B**) Correlation between *SMAD7* expression and tumor purity (**left**), and M2 macrophage infiltration level estimated using the CIBERSORT-ABS algorithm (**right**). Statistical significance and correlation strength were evaluated using Spearman’s rank correlation coefficient (Rho) and adjusted *p*-values (*p* < 0.05 considered statistically significant).

To further investigate the potential relevance of the experimental findings in human CRC, we analyzed publicly available transcriptomic data using the TIMER3 platform. SMAD7 expression showed a positive correlation with estimated total macrophage infiltration (Spearman’s ρ = 0.347, *p* = 3.19 × 10^−9^) and the estimated M2 macrophage-associated signature (Spearman’s ρ = 0.330, *p* = 2.02 × 10^−8^) (Figure 5A,B). SMAD7 expression was also negatively correlated with tumor purity (Spearman’s ρ = −0.186, *p* = 1.66 × 10^−4^), indicating that tumors with higher SMAD7 expression tended to have lower estimated tumor purity and, consequently, a greater relative contribution of non-malignant cellular components.

## 4. Discussion

The interaction between tumor metabolism and immune regulation has emerged as a fundamental determinant of cancer progression. Tumor cells not only adapt their metabolism to sustain proliferation but also modify the surrounding microenvironment through the release of metabolites and signaling molecules that influence immune cell function [16,53].

In CRC, SMAD7 expression is increased and has been associated with tumor-associated inflammatory responses and malignant features [33,35,36,37,54]. The present study extends these observations by suggesting a role for SMAD7 in tumor metabolic regulation. We demonstrate that SMAD7 inhibition reduces glycolytic activity and decreases expression of HK2, a key enzyme controlling the initial commitment of glucose to glycolysis [55]. Interestingly, glucose uptake was increased following AS-induced SMAD7 inhibition despite the reduction in glycolytic flux. This finding indicates that the decrease in glycolytic activity cannot be explained by reduced glucose availability and instead suggests a metabolic uncoupling between glucose uptake and glycolytic utilization. The concomitant reduction in HK2 expression provides a potential mechanism contributing to impaired utilization of intracellular glucose through the glycolytic pathway. However, the present study did not determine the intracellular fate of the additional glucose taken up following SMAD7 knockdown. Glucose may potentially be redirected toward alternative metabolic pathways, including the pentose phosphate pathway, glycogen synthesis, or other biosynthetic processes. Alternatively, increased glucose uptake may represent a compensatory response to reduced glycolytic utilization.

An important consideration when interpreting the present findings is the specificity of the SMAD7 inhibition approach. The SMAD7 AS oligonucleotide used in this study has been previously characterized and extensively validated by our group in multiple independent studies, demonstrating efficient inhibition of SMAD7 expression and reproducible biological effects [34,35,36,37]. In the present study, SMAD7 inhibition was confirmed at the transcript and protein levels in CRC cell lines and patient-derived organoids, and the metabolic phenotype was reproduced across HCT116 and DLD-1 cells as well as patient-derived organoids. Moreover, a reduced expression of both SMAD7 and HK2 was seen following SMAD7 silencing with a commercial siRNA, thus supporting the reproducibility of the observed phenotype. Nevertheless, we acknowledge that rescue of SMAD7 expression following AS treatment would provide an additional direct demonstration of on-target specificity.

Inflammatory signaling pathways are increasingly recognized as important regulators of cancer metabolism [56]. Cytokines such as IL-6 and IL-22, which are elevated in chronic inflammatory conditions associated with CRC development, activate STAT3 signaling and influence tumor cell survival, proliferation, and metabolic adaptation [52,57,58]. In this study, IL-6 and IL-22 stimulation enhanced p-STAT3 and HK2 expression in control CRC cells, and this response was reduced following SMAD7 inhibition. These findings, together with our previous demonstration that SMAD7 binds the STAT3 promoter and enhances STAT3 transcription [37], are consistent with a model in which SMAD7 contributes to cytokine-associated metabolic adaptation through a mechanism involving STAT3 signaling. However, the present data did not directly establish that STAT3 is required for SMAD7-dependent regulation of HK2, and additional experiments assessing STAT3 occupancy at the HK2 regulatory region will be required to establish this causal relationship. Meanwhile, the current findings should be interpreted as supporting an association between SMAD7, STAT3-related signaling, and HK2 regulation rather than demonstrating a direct causal SMAD7–STAT3–HK2 pathway.

A major finding of this study is that SMAD7-associated metabolic regulation may influence communication between CRC cells and macrophages. Tumor-associated macrophages represent a major immune component of the CRC microenvironment and contribute to tumor progression through the regulation of inflammation, extracellular matrix remodeling, angiogenesis, and immune responses [59,60,61]. Rather than representing fixed M1 or M2 populations, tumor-associated macrophages exist along a dynamic spectrum of functional states shaped by signals derived from tumor cells and the surrounding tissue environment [62,63].

Lactate has recently emerged as an important immunomodulatory metabolite within tumors [64]. Increased glycolytic activity and lactate accumulation can influence immune cell differentiation and function, promoting macrophage states associated with tissue remodeling and immune regulation [65,66]. Consistent with these observations, we found that SMAD7 inhibition reduced lactate production by CRC cells and impaired the ability of tumor cell-conditioned medium to induce the expression of macrophage-associated immunoregulatory markers, including *CD163*, *CD206*, *ARG1*, and *IL-10*, while increasing *IL-1β* expression. Importantly, supplementation with lactate restored these effects, supporting a contribution of lactate to the SMAD7-induced modulation of macrophage markers. These findings suggest that the metabolic consequences of SMAD7 inhibition may influence tumor–macrophage communication through changes in tumor-derived lactate. However, because conditioned medium contains multiple tumor-derived factors, including cytokines, growth factors, and extracellular vesicles, we do not exclude the possibility that such factors may contribute to the macrophage phenotype observed following SMAD7 inhibition.

The potential relevance of SMAD7-associated immune regulation was further explored through analysis of human CRC transcriptomic data using the TIMER3 platform. Consistent with our experimental findings, higher *SMAD7* expression was positively correlated with estimated total macrophage infiltration and with an M2 macrophage-associated signature in CRC samples. However, *SMAD7* expression was also negatively correlated with tumor purity. This observation is an important consideration when interpreting the macrophage-related associations, because tumors with lower purity contain a greater relative proportion of non-malignant cellular components, including immune and stromal cells. Thus, the positive correlations between *SMAD7* expression and macrophage-related signatures may partially reflect a broader association between *SMAD7* expression and increased non-malignant cellular infiltration rather than a macrophage-specific relationship. The present analysis was not designed to establish the prognostic significance of the proposed SMAD7–HK2–lactate pathway. Future studies integrating survival outcomes with clinically annotated cohorts and validated molecular signatures will be required to determine whether this pathway has prognostic or predictive relevance. Similarly, integration of spatial transcriptomics, single-cell sequencing, and clinical immune profiling will be important to clarify the biological significance and cellular context of the observed association.

## 5. Conclusions

This study identifies SMAD7 as a potential regulator of the metabolic–immune interface in CRC. SMAD7 inhibition is associated with reduced HK2 expression, glycolytic activity, and lactate production, which may subsequently influence macrophage phenotype within the tumor microenvironment. The attenuation of cytokine-induced HK2 expression following SMAD7 inhibition, together with previous evidence linking SMAD7 to STAT3 regulation, is consistent with a role for STAT3-related signaling in this process, although the precise molecular mechanism remains to be established. These findings expand current understanding of SMAD7 biology in CRC.

## Data Availability

The data presented in this study are available in this article and Supplementary Materials.

## Supplementary Materials

The following supporting information can be downloaded at: https://www.mdpi.com/article/10.3390/cancers18162719/s1, Figure S1: SMAD7 knockdown in DLD-1 cells regulates glycolytic activity and the expression of HK2; Figure S2: SMAD7 knockdown in HCT116 and DLD-1 cells regulates the expression of HK2; Figure S3: SMAD7 controls HK2 expression and STAT3 activation; Figure S4: SMAD7 controls HK2 expression and STAT3 activation; Figure S5: Kaplan–Meier survival analysis according to *SMAD7* expression levels.
