# Supplementary material for: SMAD7-Associated Glycolytic Regulation Promotes Lactate-Dependent Macrophage Phenotype Modulation in Colorectal Cancer
Source: Cancers (Basel). 2026 Aug 21;18(16):2719. doi: 10.3390/cancers18162719 (PMC13511421; doi:10.3390/cancers18162719)
Supplement: Supplementary file 1 [file cancers-18-02719-s001.zip › cancers-4456178-original WBs.pdf]

Supplementary Material Colella etal.;

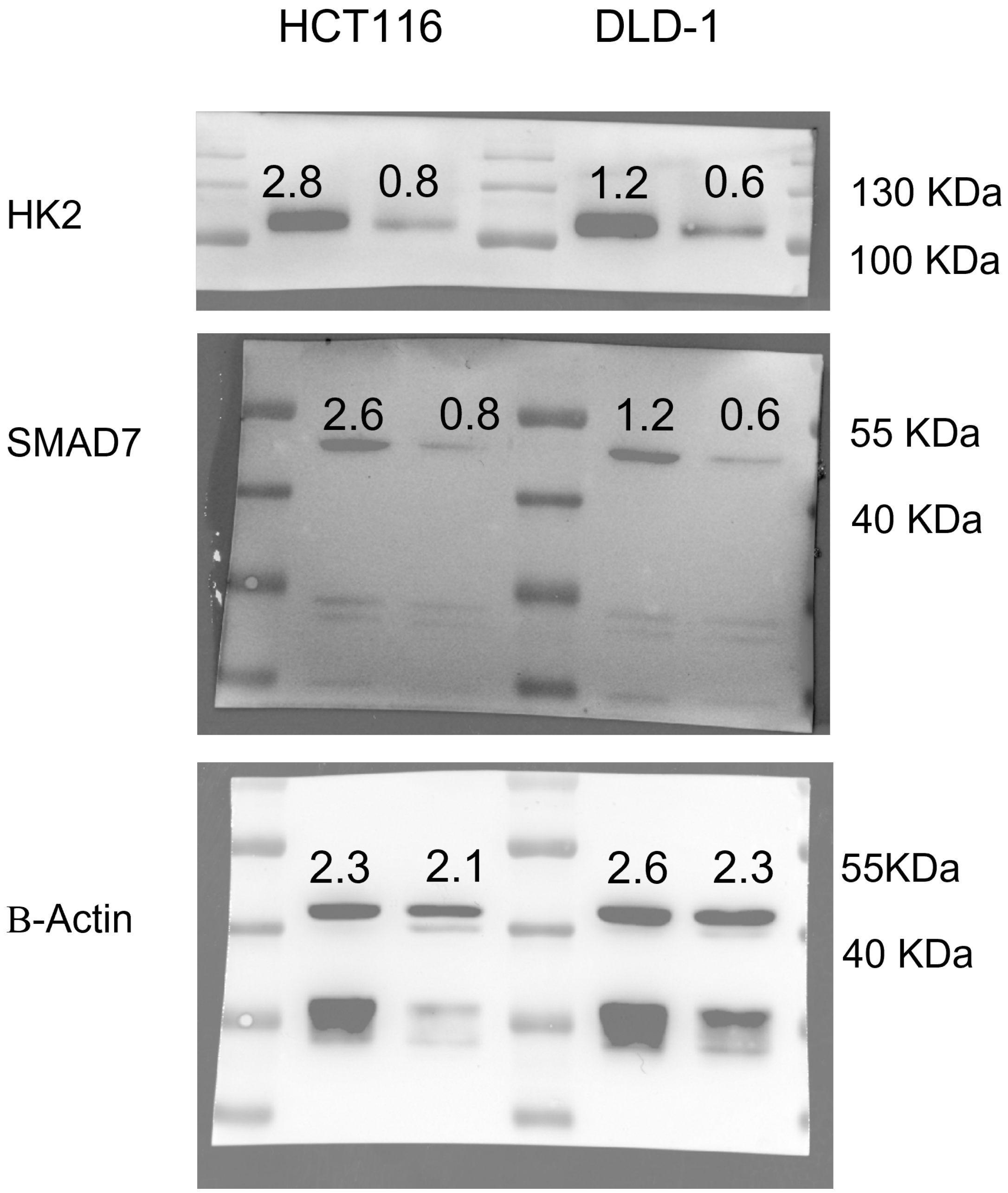

Uncropped images of figure 1 and figure S1.

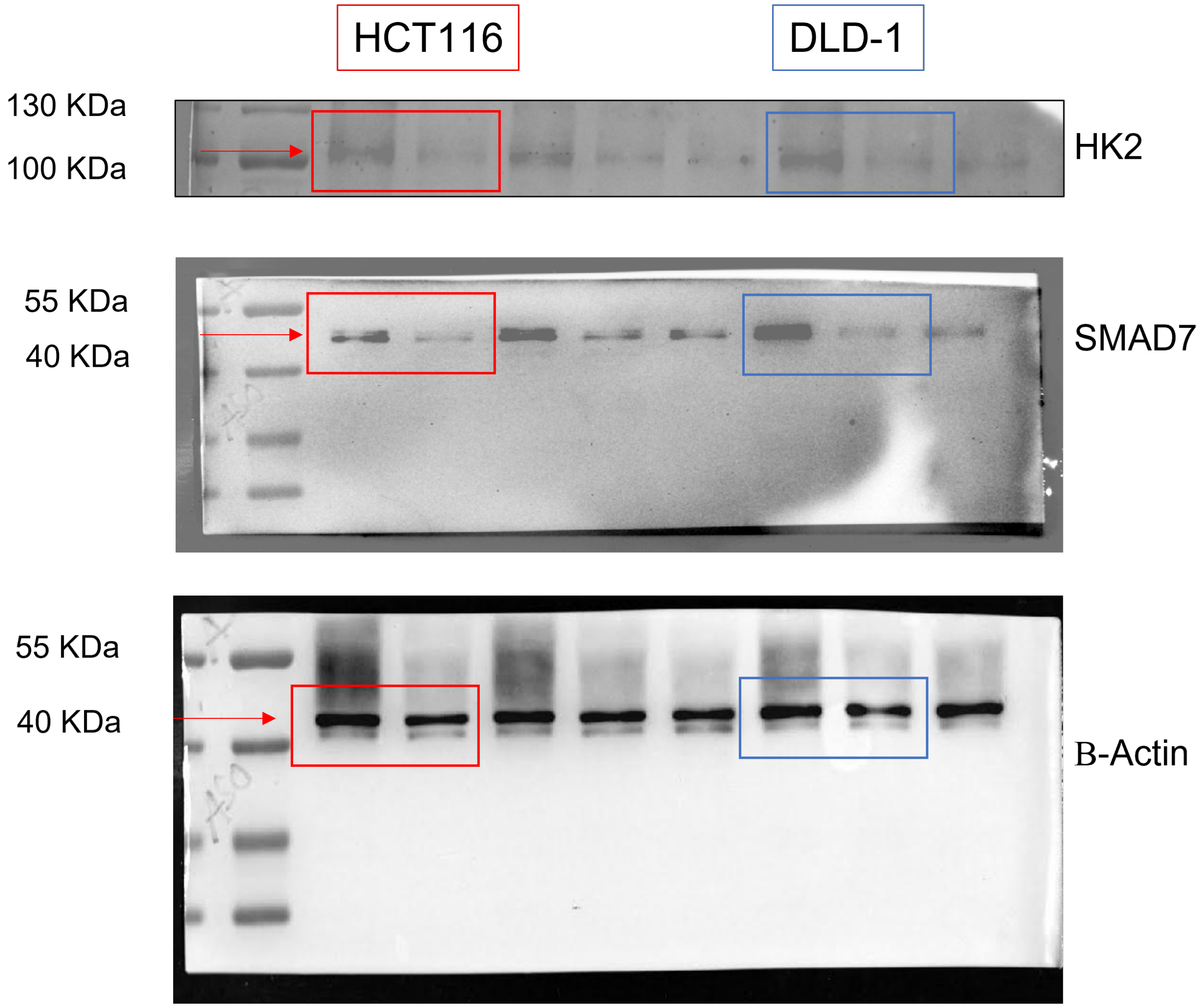

Uncropped images of figures S2

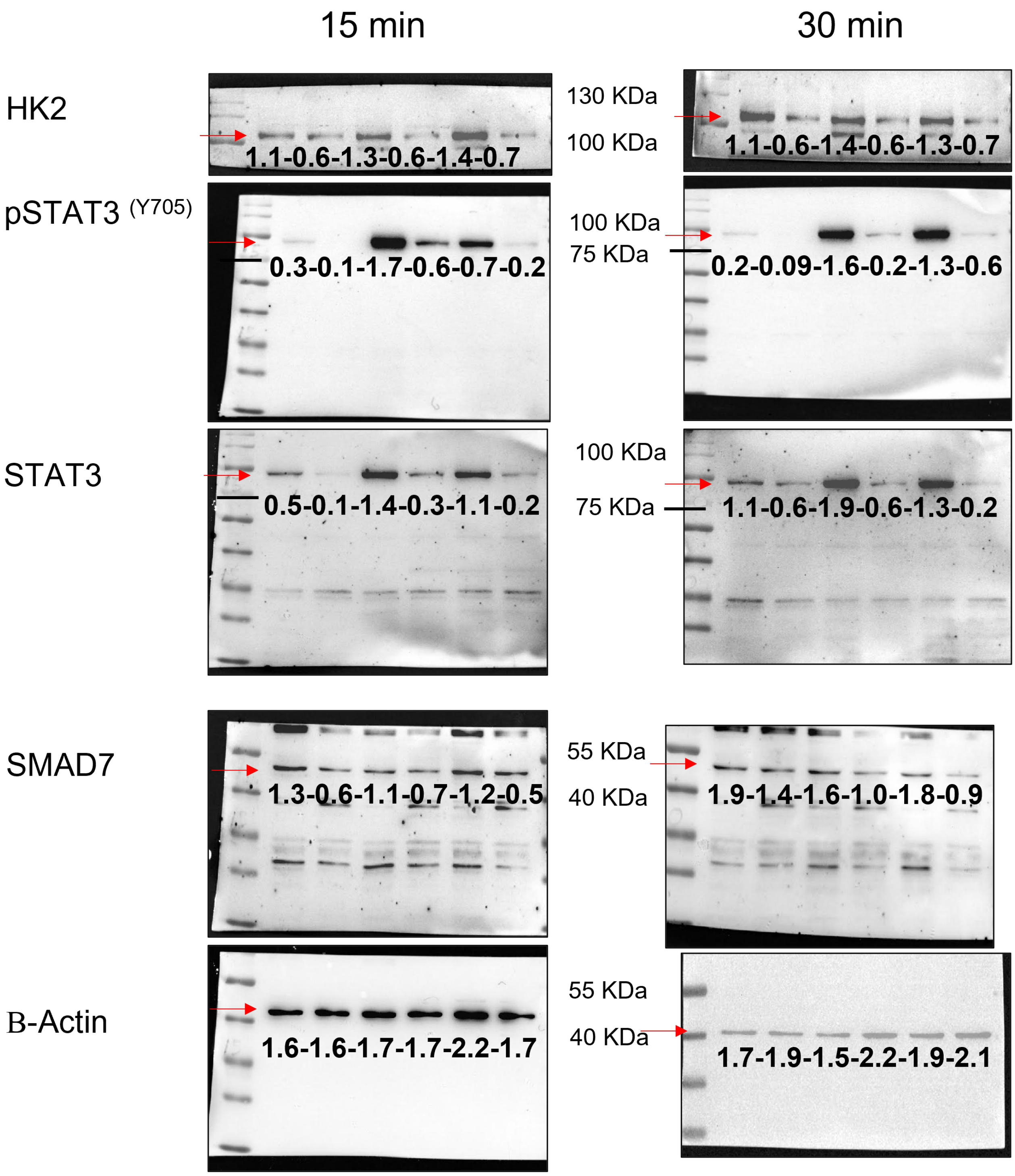

Uncropped images of figure 3

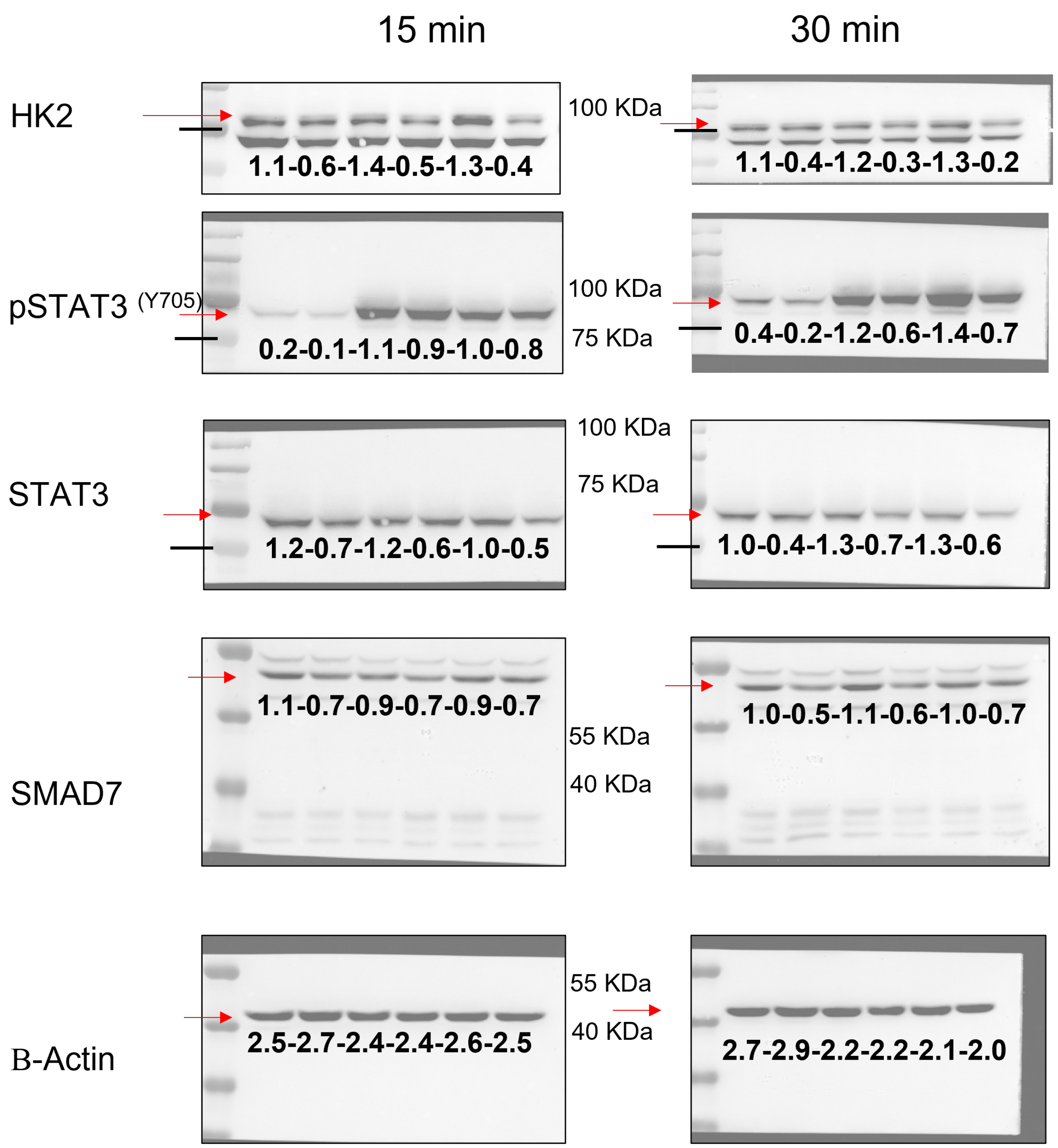

Uncropped images of figure S4
