# Supplementary material for: SMAD7-Associated Glycolytic Regulation Promotes Lactate-Dependent Macrophage Phenotype Modulation in Colorectal Cancer
Source: Cancers (Basel). 2026 Aug 21;18(16):2719. doi: 10.3390/cancers18162719 (PMC13511421; doi:10.3390/cancers18162719)
Supplement: Supplementary file 1 [file cancers-18-02719-s001.zip › cancers-4456178-suppl-proof.pdf]

Supplementary Materials

SMAD7-Associated Glycolytic Regulation Promotes Lactate-Dependent Macrophage Phenotype Modulation in Colorectal Cancer

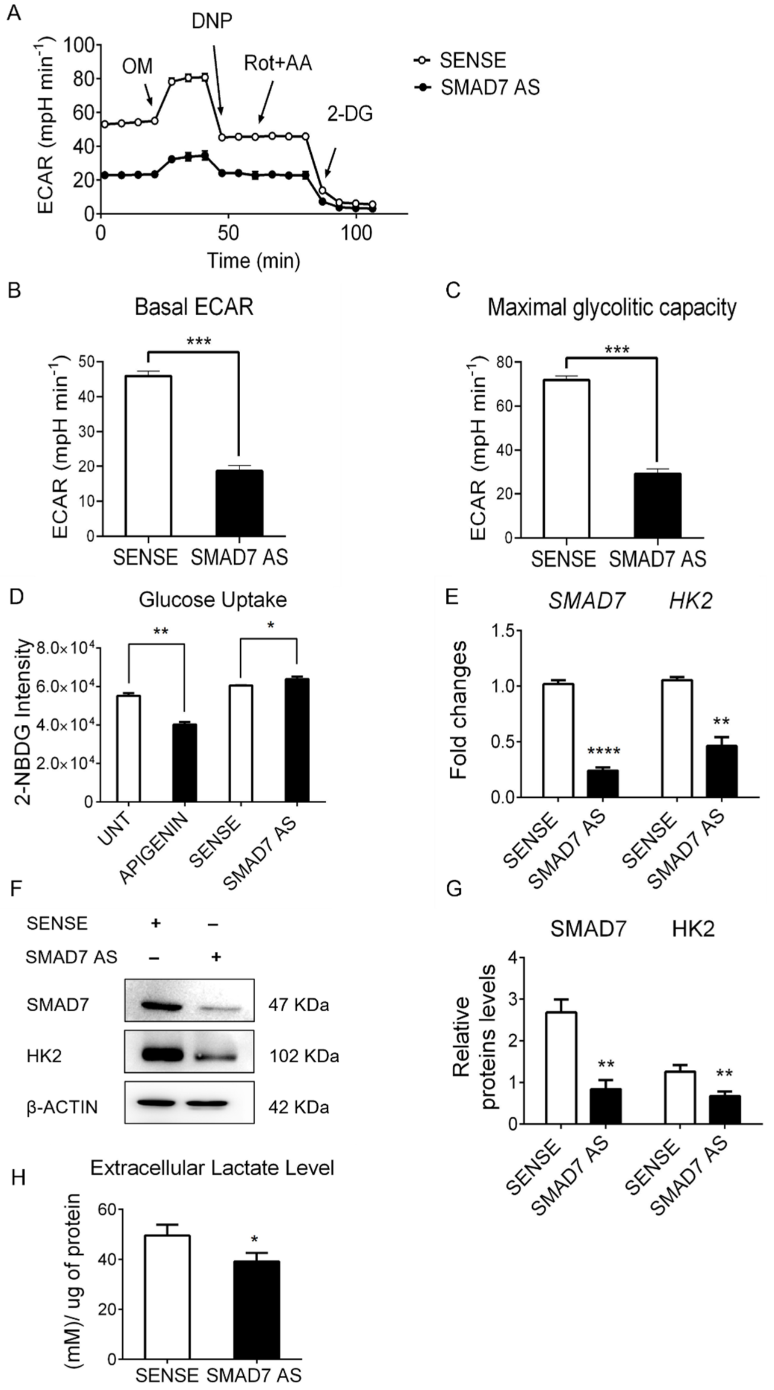

Figure S1. SMAD7 knockdown in DLD-1 cells regulates glycolytic activity and the expression of HK2. (**A**) Extracellular acidification rate (ECAR) were measured using a Seahorse Assay. (**B,C**) Quantification of metabolic parameters basal glycolysis and maximal glycolytic capacity are

reported. (OM: Oligomycin; DNP: 2,4 Dinitrophenol; Rot: Rotenone; AA: Antimycin A; 2-DG: 2-deoxyglucose). Values show the mean ± SD of three independent experiments. Differences were analyzed using a two-tailed Student's *t*-test (* $p < 0.05$, (** $p < 0.01$). (**D**) Glucose Uptake evaluated in SMAD7-deficient cells. Values show the mean ± SD of three independent experiments. Differences were analyzed using a two-tailed Student's *t*-test (* $p < 0.05$, *** $p < 0.001$). (**E**) DLD-1 cells were transfected with either SMAD7 sense or AS for 24 h and *SMAD7* and *HK2* mRNA transcripts were evaluated by real-time polymerase chain reaction. Levels were normalized to B2M. Values show the mean ± SD of three independent experiments. Differences were analyzed using a two-tailed Student's *t*-test (**** $p < 0.0001$). (**F**) Cells were transfected, as indicated in M&M section, and SMAD7 and HK2 proteins were analyzed by Western blotting. Panel (**G**) shows the quantitative analysis of SMAD7, HK2, and β-Actin, as evaluated by the densitometry scanning of Western blots. Values indicate the mean ± SD of three independent experiments; differences were analyzed using a two-tailed Student's *t*-test (** $p < 0.01$, *** $p < 0.001$). (**H**) Quantification of extracellular lactate levels in DLD-1 cells line. Values indicate the mean ± SD of three independent experiments; differences were analyzed using a two-tailed Student's *t*-test (* $p < 0.05$).

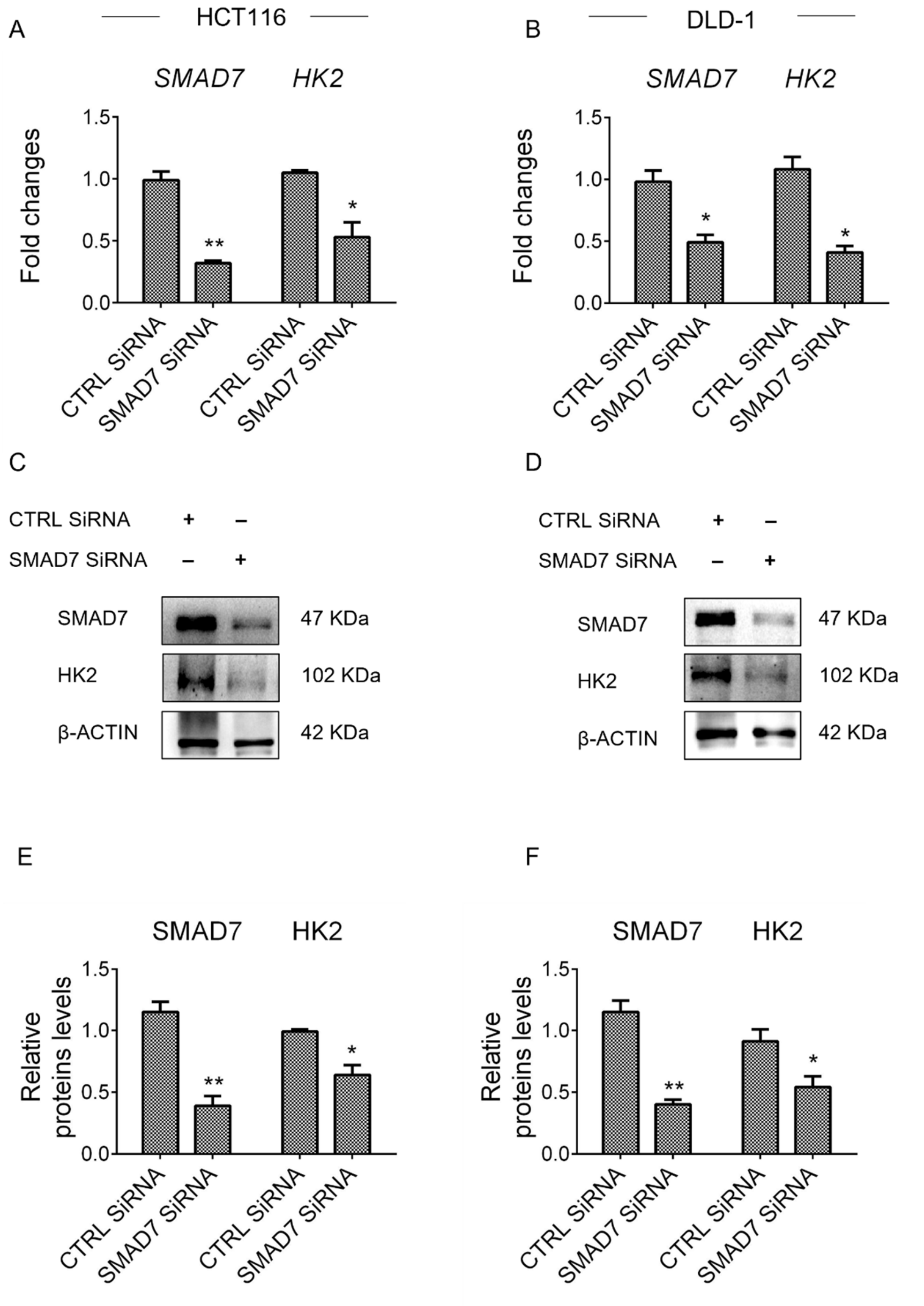

Figure S2. SMAD7 knockdown in HCT116 and DLD-1 cells regulates the expression of HK2. (**A**) HCT116 and DLD-1 (**B**) cells were transfected with either CTRL SiRNA (10nM) or SMAD7 SiRNA (10 nM) for 24 h, and then *SMAD7* and *HK2* RNA transcripts were evaluated by real-time polymerase chain reaction. Levels were normalized to *B2M*. Values show the mean ± SD of three independent experiments. Differences were analyzed using a two-tailed Student's *t*-test (* $p < 0.05$; ** $p < 0.01$). (**C**) HCT116 and DLD-1 (**D**) cells were transfected as reported above, and SMAD7, HK2 and β-Actin proteins were analyzed by Western blotting. (**E,F**) Panels show quantitative analysis of SMAD7 and HK2 as evaluated by densitometry of Western blots. Levels were normalized to β-Actin. Values indicate the mean ± SD of three independent experiments; differences were analyzed using a two-tailed Student's *t*-test (* $p < 0.05$, ** $p < 0.01$).

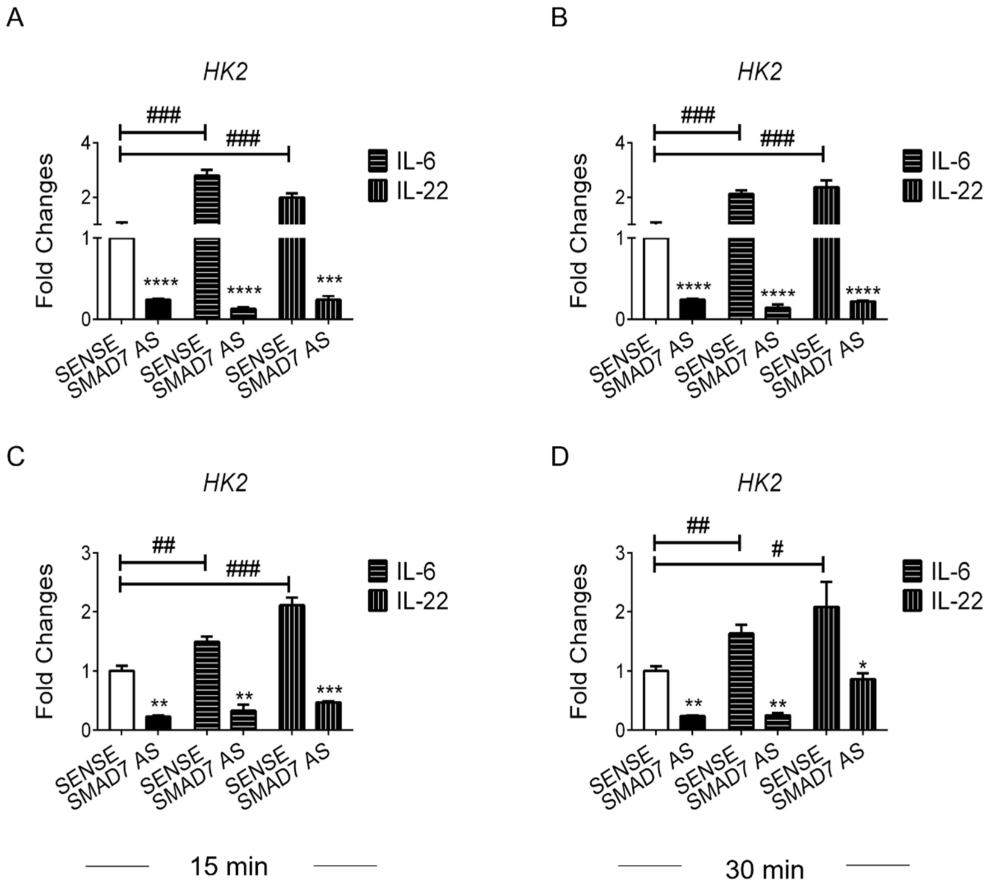

Figure S3. SMAD7 controls HK2 expression and STAT3 activation. (**A,B**) HCT116 and DLD-1 (**C,D**) cells were transfected with either SMAD7 sense or AS for 18 h, and then either left untreated or stimulated with IL-6 or IL-22 for 15 or 30 minutes. *HK2* RNA was evaluated by real-time polymerase chain reaction. Levels were normalized to *B2M*. Differences were analyzed using a two-tailed Student's *t*-test (** $p < 0.01$,*** $p < 0.001$, **** $p < 0.0001$; $^{\#}$ $p < 0.05$, $^{\#\#}$ $p < 0.01$, $^{\#\#\#}$ $p < 0.001$).

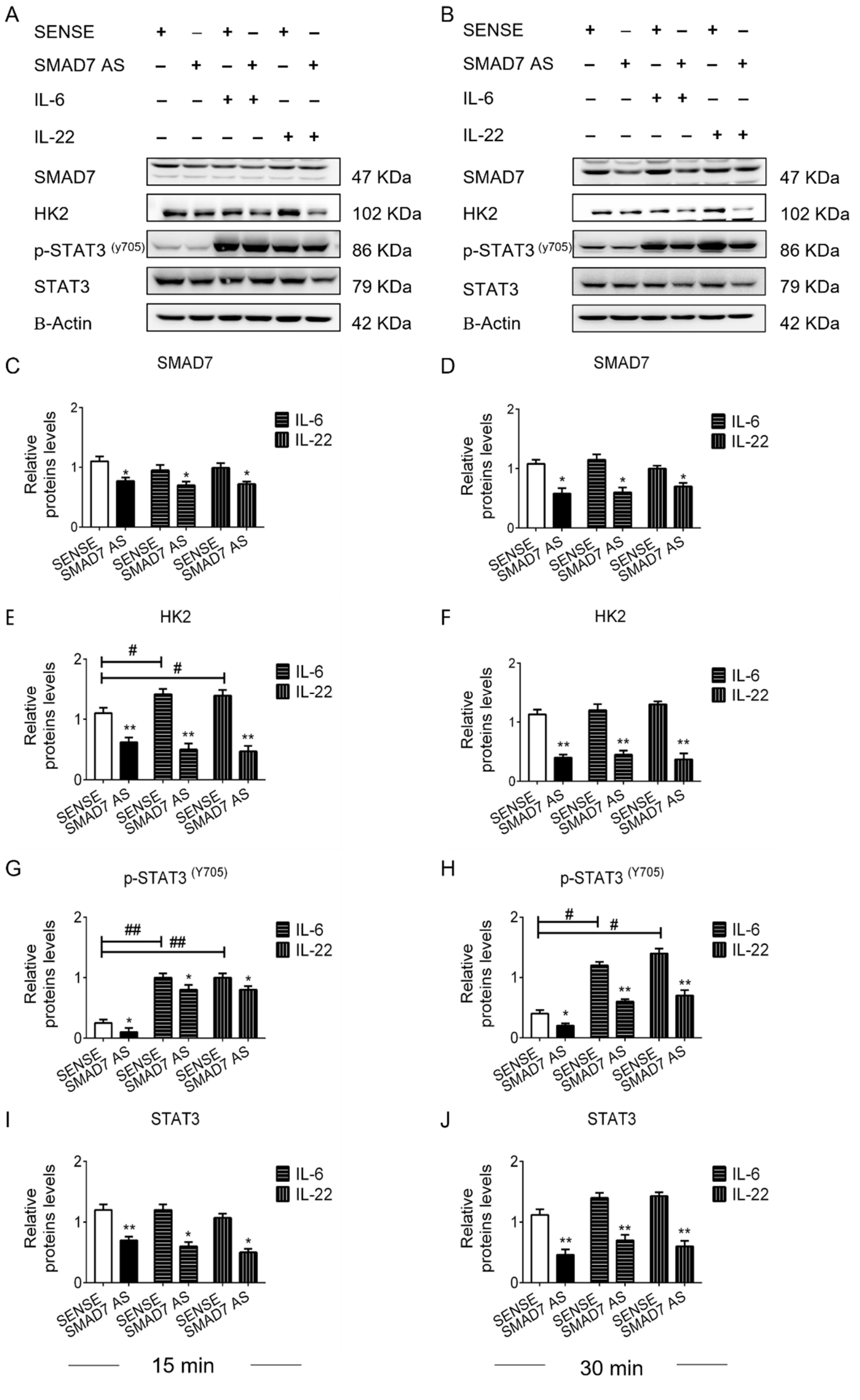

Figure S4. SMAD7 controls HK2 expression and STAT3 activation. (**A**,**B**) Cells were transfected with either SMAD7 sense or AS for 24 h, and then either left untreated or stimulated with IL-6 or Il-22 for 15 or 30 minutes. SMAD7, HK2, p-STAT3$^{(y705)}$, STAT3 and β-Actin proteins were analyzed by Western blotting. Panel (**C**–**J**) shows the quantitative analysis of SMAD7, HK2, p-STAT3$^{(y705),}$ and STAT3, as evaluated by densitometry of Western blots. Levels were normalized to β-Actin. Values indicate the mean ± SD of three independent experiments; Differences were analyzed using a two-tailed Student's *t*-test (* $p < 0.05$, ** $p < 0.01$, $^{\#}$ $p < 0.05$, $^{\#\#}$ $p < 0.01$).

A

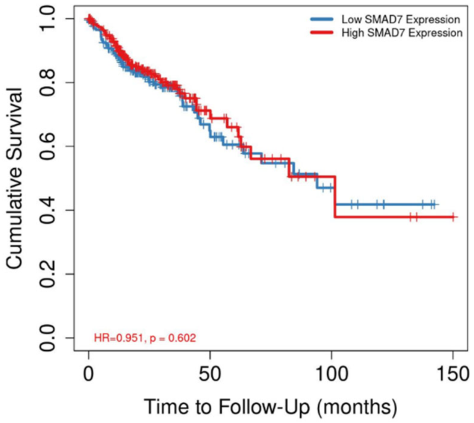

Figure S5. Kaplan-Meier survival analysis according to *SMAD7* expression levels. Overall survival (OS) curves for patients stratified by high (red line) and low (blue line) *SMAD7* gene expression. Differences between groups were evaluated using the log-rank test (HR = 0.951$, *p* = 0.602). Follow-up time is shown in months.
